# Psychological distress among healthcare students in Poland from COVID-19 to war on Ukraine: a cross-sectional exploratory study

**DOI:** 10.3389/fpubh.2023.1186442

**Published:** 2023-06-19

**Authors:** Tomomi Hisato, Shreya Nandy, Eman M. Monga, Paulina Sytek, Mohamed Abouzid, Alhassan Ali Ahmed

**Affiliations:** ^1^Faculty of Medicine, Poznan University of Medical Sciences, Poznan, Poland; ^2^Department of Dental Surgery, Poznan University of Medical Sciences, Poznan, Poland; ^3^Department of Physical Pharmacy and Pharmacokinetics, Faculty of Pharmacy, Poznan University of Medical Sciences, Poznan, Poland; ^4^Doctoral School, Poznan University of Medical Sciences, Poznan, Poland; ^5^Department of Bioinformatics and Computational Biology, Poznan University of Medical Sciences, Poznan, Poland

**Keywords:** war, anxiety, depression, stress, medical students, psychache, COVID-19, pandemic

## Abstract

**Objectives:**

Healthcare students went through a rough time in March 2022 due to extreme changes in the educational system (moving from online to stationary learning) and Ukrainian-Russian war circumstances. Our study aims to update knowledge about psychological distress and its impact on healthcare students in Poland after two years of the COVID-19 pandemic, followed by intense and political instability in Europe.

**Methods:**

We conducted a cross-sectional study on healthcare students from Poznan University of Medical Sciences, Poland, from March to April 2022. The questionnaire included subjective retrospective 5-point Likert-scales ratings of anxiety, stress, and depression and self-reported information on various psychological distress predictors.

**Results:**

The anxiety levels at the beginning of the COVID-19 pandemic were higher than in April 2022. There was no significant reduction in stress and depression. Females had higher initial anxiety levels than post-pandemic levels. Higher reported levels of anxiety, stress, and depression were significantly correlated with political instability in Eastern Europe (Spearman r_anxiety_ = 0.178, r_stress_ = 0.169, r_depression_ = 0.154, *p* ≤ 0.001, respectively). The concern about moving towards online education showed a significant association only with stress level (r_stress_ = 0.099, *p* = 0.034). We also observed a positive correlation between anxiety, stress, and depression and deteriorating sleep quality (Spearman r_anxiety_,=0.325, r_stress_ = 0.410, r_depression_ = 0.440, *p* < 0.001), the feeling of worsening relationships with family and peers (r_anxiety_ = 0.325, r_stress_ = 0.343, r_depression_ = 0.379, *p* < 0.001), and the sense of loss of efficient time management (r_anxiety_ = 0.321, r_stress_ = 0.345, r_depression_ = 0.410, p < 0.001).

**Conclusion:**

Throughout the progression of the Ukrainian war and the COVID-19 pandemic, females reported improved (lower levels) levels of anxiety. Nevertheless, the current levels of self-reported anxiety post-pandemic remain alarming, while stress and depression levels remained unchanged. Mental, psychological, and social support activities are required for healthcare students, especially those away from their families. Time management, academic performance, and coping skills in relation to the additional stressors of war and the global pandemic require further research in this group of students.

## Graphical Abstract

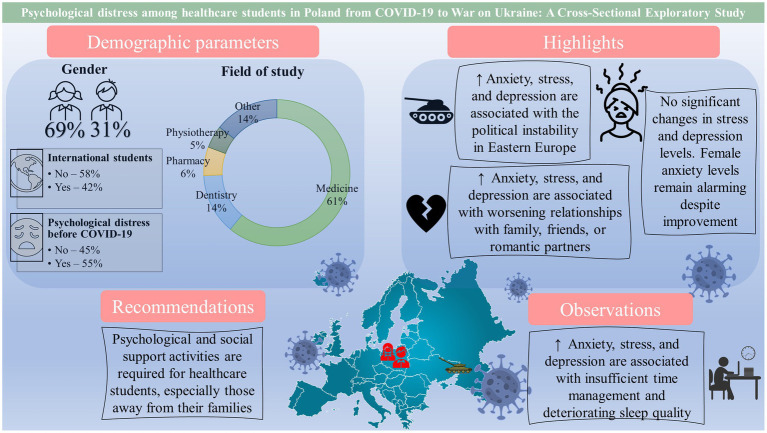

## 1. Introduction

**Figure 1:**
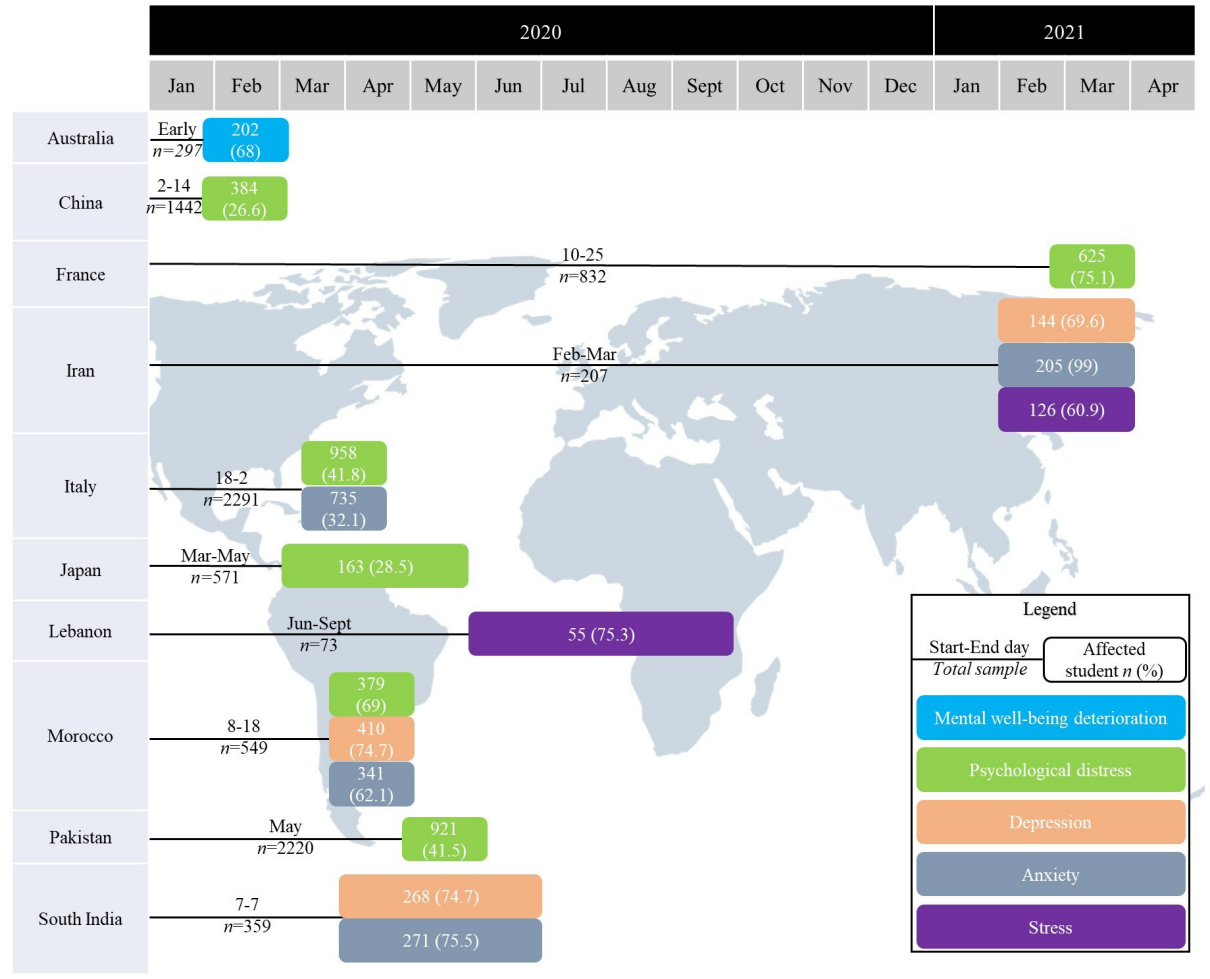
Signs of psychological distress, depression, anxiety, stress, and mental well-being deterioration for healthcare students worldwide.

The World Health Organisation (WHO) declared the pandemic of coronavirus disease 2019 (COVID-19) in March 2020 (1). Governments worldwide started implementing strict restrictions to limit the spread of the virus and prevent an overload on the healthcare system. The pandemic and accompanying restrictions led to increased psychological distress, anxiety, and depression in the general population (2, 3), however, the specific impact on healthcare students in eastern Europe has inadequately been studied. On March 12, 2020, eight days after the first COVID-19 case was identified in Poland, the national government introduced a closure of all academic institutions, leading to a rise in anxiety and perceived stress among many students (4). Medical students in Poland increased their consumption of alcohol, cigarettes, and other stimulants, and 40% showed the intention to reach out to a psychiatrist or psychologist for their deteriorating mental health caused by the pandemic (5). On the other hand, many medical students engaged in additional work during the pandemic and perceived this as beneficial (6). Globally, 50 % of individuals reported psychological distress during the COVID-19 pandemic in the general population (7). Psychological distress is defined as “non-specific symptoms of stress, anxiety, and depression” (8). It can negatively affect the quality of the individual’s studies, relationships, sleep, and any components of life (9). The percentage varied between countries and depended on their respective healthcare systems’ strength and the restrictions imposed (7). Healthcare students worldwide showed signs of psychological distress, which depended on: perceived stress, amount of time spent following the pandemic news, frequency of pandemic-related dreams, and perceived social support (10–20) (Figure 1).

While the world was struggling with the following waves of the pandemic, other countries were dealing with added external stresses, such as the Russian invasion of Ukrainian, including cities close to Polish borders on February 24, 2022, and elevated the political strain in Europe (21). More than 3 million Ukrainian refugees migrated to Poland within a short time, mostly women and children. This has caused a necessity to establish refugee centers and caused additional challenges on political, economic, social, and public health levels (22, 23) Historically, Poles, like Ukrainians, has been concerned about the threat of a Russian invasion. According to a recent study on Polish citizens (*N* = 110), approximately 53% of participants experienced an increase in both sadness and depression since the outbreak of the war in Ukraine reported (24).

In the early phases of COVID-19 between March and April 2020, 68–75% of the Polish population reported a higher level of stress (25–27), however few studies reflect on the more recent phases of the pandemic. One study conducted in early 2022 reported that 73% suffered from burnout, but not many studies reflect on the more recent phases of the pandemic (28). With a focus on students, studies showed an increased prevalence of psychological distress during the highly restrictive period of the pandemic and reported 56% of high levels of stress between March and April 2020 (29) and 76.96% of manifested psychological symptoms between May and June 2020 (30). Studies concerning medical students in Poland solely reported, stress-coping reactions, or quality of life during the COVID-19 pandemic leaving psychological distress levels among medical students in Poland largely unstudied (5, 31, 32). The student population studied herein experienced the end of the COVID-19 pandemic restrictions thus, are expected to be well-adapted since the lift of restrictions on 28 March 2022 (33). The pandemic has served as a huge factor in the psychological distress seen in many students, however, external political stresses from the Russian invasion of Ukraine have also played a part since February 2022 (21). We hypothesized that the psychological distress experienced during the last phase of the pandemic would be different from that during the early phases due to an increase in other stress-inducing factors.

This study aimed to analyze how COVID-19 impacted the mental health of healthcare students in Poland from different disciplines of the healthcare field (i.e., medical, dental, pharmacy, physiotherapy). We explored the effects of the pandemic by assessing mental health at a predetermined baseline, taking into account various factors such as individual course load, year of study, and external stress-inducing factors (i.e., war, social interactions, public settings). We study the initial (when COVID-19 was first discovered) and current (post-pandemic or during the time of study March–April 2022) in order to provide a suggested guideline for necessary supportive resources at academic institutions for students combating mental health challenges.

This study would therefore address the following research questions by collecting the data retrospectively at one point in time:

- What are the differences between anxiety, stress, and depression levels from November 2019 (the beginning of COVID-19) and April 2022?
- What is the impact of demographic parameters (gender, year of study, field of study, weekly teaching hours, pre-pandemic psychological distress, and being an international student) on the levels of anxiety, stress, and depression among healthcare students?
- What are the correlations between psychological distress predictors and anxiety, stress and depression?

## 2. Methods

### 2.1. Study Design

We performed a cross-sectional study following the Strengthening the Reporting of Observational Studies in Epidemiology (STROBE) guidelines (Supplementary File 1) (34). We used an anonymous, self-administered online survey tool through the “Microsoft Forms” platform.

### 2.2. Study population

The inclusion criteria for individuals who consented to participate in the study included: age ≥ 18 years and being a student at Poznan University of Medical Sciences (PUMS). There were no restrictions on gender, nationality, or socioeconomic level. The exclusion criteria were all participants less than 18 years who refused to participate in the study or inaccurately filled the survey.

### 2.3. Sampling

The survey was distributed in March–April 2022 using student groups. We randomly sampled individuals utilizing the PUMS library, whereby consenting students filled out the surveys voluntarily. Researchers took shifts at the library based on their schedules and were usually done by the hour. Our other method of student recruitment was reaching out to student organizations and utilizing multiple social media platforms. OpenEpi was used to calculate the sample size. Assuming there are 1,218,000 million students in Poland and 11.3% met the inclusion criteria, 95% confidence intervals, and 5% confidence limit. The estimated minimum sample size was 154.

### 2.4. Study tool

We divided our survey into three sections: (1) the demographic characteristics (age, gender, the field of study), year of study, course load since COVID-19 started, if the participant is an international student or not, if the participant had struggled with psychological distress before the COVID-19 pandemic; (2) assess the level of anxiety, stress, and depression measured on a 1–5 scale of normal, mild, moderate, severe and extremely severe; (3) predictors of psychological distress (current political situation in Eastern Europe during COVID-19) such as participant’s career, remote education concerns, family concerns, inability to back again to everyday life and character as before pandemic, mental exhaustion, the need for psychological support, the impact of staying at home for a long time, sleep quality, participation in sports, personal relationships, the impact of remote learning and time management. The survey is included in the Supplementary materials (Supplementary File 2, questionnaire). The scales’ internal consistency reliability was determined with Cronbach’s standardized alpha and demonstrated good reliability of α = 0.796 (35). The survey validation file is attached in Supplementary materials (Supplementary File 3, questionnaire validation).

### 2.5. Ethical consideration

We conducted this study according to the Declaration of Helsinki (36). Written informed consent was obtained from all participants. This study did not require ethical approval because it was not a clinical trial but was a surveillance of the population’s attitude. The Bioethics Committee of Poznan University of Medical Sciences has issued the ethical waiver (KB-483/22).

### 2.6. Statistical analysis

We performed the statistical analysis using Statistica version 13 coupled with Plus Kit version 3. The pairwise deletion was used for missing data. Shapiro–Wilk test was used to measure the normality of continuous data. Categorical data were reported as frequency/percentage and continuous data as mean/standard deviation (SD) (for normal distribution and Scales of anxiety, stress, and depression) or median/interquartile range (IQR) (for non-normal distribution). Intra-differences between variables (i.e., initial anxiety levels *vs*. current anxiety levels for females) were calculated using a non-parametric Wilcoxon signed-rank test. Mann–Whitney *U* test measured the differences of anxiety, stress, or depression between females and males. Chi-square with Bonferroni correction was used to measure the inter-differences between the groups (i.e., initial anxiety levels between males vs. females). Moreover, Spearman’s rank correlation coefficient (r) was used to investigate the correlation between several predictors; and between psychological distress predictors and stress, depression, and anxiety. A *p*-value less than 0.05 was statistically significant in all tests.

## 3. Results

### 3.1. Demographic characteristics of the participants

A total of 461 participants filled out the survey among whom 69.2% were female. The age range was 22.36 ± 3.02: [22 (20–24)]. Most participants studied medicine, followed by dentistry, pharmacy, and physiotherapy. The maximum number of students reported studying more than 20 h per week. Demographically, 57.5% of participants were Polish nationals, and 42.5% were international students. Almost 55.1% of participants reported psychological distress before the COVID-19 pandemic (due to work-related stress, anxiety, or personal issues). Demographic statistics are presented in Table 1.

**Table 1:** Demographic data of the participants, frequencies reported as *N* (%).

Age	22.36 ± 3.02; [22 (20–24)]^*^
Gender	
Female	319 (69.2)
Male	142 (30.8)
Year of study	2.82 ± 1.70; [3 (1–4)]^*^
< 4 years	320 (69.4)
≥ 4 years	141 (30.6)
Field of study	
Medicine	281 (61)
Dentistry	62 (13.4)
Pharmacy	29 (6.3)
Physiotherapy	23 (5)
Other	66 (14.3)
Weekly teaching hours	
5 to <10 h a week	59 (12.8)
10 to <15 h a week	130 (28.2)
15 to <20 h a week	121 (26.2)
≥ 20 h a week	151 (32.8)
Psychological distress before COVID-19	
No	207 (44.9)
Yes	254 (55.1)
International students	
No	265 (57.5)
Yes	196 (42.5)

^*^reported as mean ± SD; [median (IQR)].

### 3.2. Initial and current levels of anxiety, stress, and depression

The average initial anxiety level was higher than the current level in the PUMS student population (2.37 ± 1.13 vs. 2.19 ± 1.17, *p* = 0.006); there were no significant differences in overall stress and depression.

Female students had higher initial compared to current anxiety levels (2.43 ± 1.14 vs. 2.25 ± 1.16, *p* = 0.021). Males reported numerically lower post-versus pre-pandemic anxiety levels, however, the results were statistically insignificant (2.23 ± 1.12 vs. 2.05 ± 1.17, *p* = 0.146). There was no significant difference between males and females in anxiety, stress, or depression. Dentistry students had higher initial versus current anxiety levels (2.73 ± 1.03 vs. 1.87 ± 0.97, *p* < 0.001), students with weekly courseload from 10 to <15 h (2.42 ± 1.08 vs. 2.15 ± 1.19, *p* = 0.024), students with weekly courseload from 15 to <20 h (2.55 ± 1.13 vs. 2.13 ± 1.05, *p* = 0.002), senior students (2.46 ± 1.14 vs. 2.05 ± 1.09, *p* = 0.001), students who declared no psychological distress before the pandemic (2.00 ± 0.99 vs. 1.69 ± 0.98, p = 0.001), and non-international students (2.38 ± 1.13 vs. 2.16 ± 1.14, *p* = 0.015). Students with weekly courseload from 15 to <20 h presented increased initial stress levels compared to the current level (2.62 ± 1.2 vs. 2.35 ± 1.27, *p* = 0.043), as well as senior students (2.4 ± 1.12 vs. 2.13 ± 1.14, *p* = 0.025) and students who declared no psychological distress before the pandemic (2.06 ± 1.06 vs. 1.87 ± 1.05, *p* = 0.034). Students with weekly courseload ≥20 h experienced had lower initial depression level than the current level (2.08 ± 1.15 vs. 2.39 ± 1.22, *p* = 0.007; Table 2).

**Table 2:** Intra-difference analysis between initial and current levels of anxiety, stress, and depression using Paired Wilcoxon Signed-Rank Test.

Parameter		Initial level		Current level		*Z*	*p*
		Mean (SD)	Median (Q1-Q3)	Mean (SD)	Median (Q1-Q3)		
Anxiety							
Gender	Female	2.43 (1.14)	2 (1–3)	2.25 (1.16)	2 (1–3)	2.31	0.021
	Male	2.23 (1.12)	2 (1–3)	2.05 (1.17)	2 (1–3)	1.45	0.146
Field of study	Medicine	2.31 (1.14)	2 (1–3)	2.2 (1.15)	2 (1–3)	1.34	0.180
	Dentistry	2.73 (1.03)	3 (2–3)	1.87 (0.97)	2 (1–3)	4.33	<0.001
	Pharmacy	2.03 (0.82)	2 (1–3)	2.14 (1.3)	2 (1–3)	0.24	0.808
	Physiotherapy	2.52 (1.24)	3 (1–3)	2.48 (1.44)	2 (1–4)	0.24	0.811
	Other	2.38 (1.24)	2 (1–3)	2.33 (1.23)	2 (1–3)	0.15	0.877
Weekly course load (hrs.)	5 to <10	2.12 (1.1)	2 (1–3)	1.93 (1.06)	1 (1–3)	1.04	0.296
	10 to <15	2.42 (1.08)	2 (2–3)	2.15 (1.19)	2 (1–3)	2.26	0.024
	15 to <20	2.55 (1.13)	2 (2–3)	2.13 (1.05)	2 (1–3)	3.10	0.002
	≥20	2.28 (1.18)	2 (1–3)	2.36 (1.26)	2 (1–3)	0.63	0.526
Year of study (grade)	< 4	2.33 (1.13)	2 (1–3)	2.25 (1.19)	2 (1–3)	1.08	0.281
	≥ 4	2.46 (1.14)	2 (2–3)	2.05 (1.09)	2 (1–3)	3.21	0.001
Psychological distress before COVID-19	No	2 (0.99)	2 (1–3)	1.69 (0.98)	1 (1–2)	3.22	0.001
	Yes	2.67 (1.15)	3 (2–4)	2.59 (1.15)	3 (2–3)	0.89	0.375
International students	No	2.38 (1.13)	2 (1–3)	2.16 (1.14)	2 (1–3)	2.44	0.015
	Yes	2.35 (1.14)	2 (1–3)	2.22 (1.2)	2 (1–3)	1.29	0.197
Total		2.37 (1.13)	2 (1–3)	2.19 (1.17)	2 (1–3)	2.73	0.006
Stress							
Gender	Female	2.51 (1.17)	2 (2–3)	2.39 (1.23)	2 (1–3)	1.54	0.123
	Male	2.3 (1.11)	2 (1–3)	2.2 (1.19)	2 (1–3)	0.77	0.444
Field of study	Medicine	2.46 (1.16)	2 (1–3)	2.39 (1.2)	2 (1–3)	0.88	0.378
	Dentistry	2.39 (1.05)	2 (2–3)	2.06 (1.17)	2 (1–3)	1.84	0.066
	Pharmacy	2.34 (1.14)	2 (1–3)	2.03 (1.21)	2 (1–3)	1.11	0.266
	Physiotherapy	2.43 (1.08)	2 (2–3)	2.57 (1.24)	3 (1–4)	0.47	0.638
	Other	2.48 (1.32)	2 (1–3)	2.38 (1.32)	2 (1–3)	0.53	0.595
Weekly course load (hrs.)	5 to <10	2.39 (1.11)	2 (1–3)	2.12 (1.08)	2 (1–3)	1.85	0.064
	10 to <15	2.37 (1.11)	2 (1–3)	2.28 (1.19)	2 (1–3)	0.76	0.449
	15 to <20	2.62 (1.2)	3 (2–3)	2.35 (1.27)	2 (1–3)	2.02	0.043
	≥20	2.4 (1.17)	2 (1–3)	2.45 (1.25)	2 (1–3)	0.48	0.634
Year of study (grade)	< 4	2.47 (1.17)	2 (1–3)	2.42 (1.25)	2 (1–3)	0.58	0.565
	≥ 4	2.4 (1.12)	2 (1–3)	2.13 (1.14)	2 (1–3)	2.24	0.025
Psychological distress before COVID-19	No	2.06 (1.06)	2 (1–3)	1.87 (1.05)	1 (1–3)	2.12	0.034
	Yes	2.76 (1.14)	3 (2–4)	2.7 (1.22)	3 (2–4)	0.49	0.627
International students	No	2.42 (1.14)	2 (1–3)	2.32 (1.2)	2 (1–3)	1.26	0.209
	Yes	2.48 (1.19)	2 (1–3)	2.35 (1.25)	2 (1–3)	1.19	0.236
Total		2.45 (1.16)	2 (1–3)	2.33 (1.22)	2 (1–3)	1.72	0.086
Depression							
Gender	Female	2.3 (1.24)	2 (1–3)	2.26 (1.18)	2 (1–3)	0.20	0.843
	Male	2.23 (1.26)	2 (1–3)	2.28 (1.22)	2 (1–3)	0.55	0.580
Field of study	Medicine	2.19 (1.21)	2 (1–3)	2.31 (1.16)	2 (1–3)	1.53	0.126
	Dentistry	2.4 (1.26)	2.5 (1–3)	2.03 (1.21)	1.5 (1–3)	1.87	0.061
	Pharmacy	2.03 (1.09)	2 (1–3)	2.07 (1.07)	2 (1–3)	0.02	0.984
	Physiotherapy	2.39 (1.2)	3 (1–3)	2.43 (1.12)	2 (1–3)	0.10	0.918
	Other	2.58 (1.41)	2 (1–4)	2.35 (1.38)	2 (1–3)	1.03	0.305
Weekly course load (hrs.)	5 to <10	2.36 (1.36)	2 (1–3)	2.07 (1.1)	2 (1–3)	1.53	0.126
	10 to <15	2.35 (1.23)	2 (1–3)	2.32 (1.2)	2 (1–3)	0.16	0.874
	15 to <20	2.4 (1.3)	2 (1–3)	2.16 (1.19)	2 (1–3)	1.68	0.093
	≥20	2.08 (1.15)	2 (1–3)	2.39 (1.22)	2 (1–3)	2.69	0.007
Year of study (grade)	< 4	2.22 (1.19)	2 (1–3)	2.33 (1.22)	2 (1–3)	1.38	0.169
	≥ 4	2.4 (1.35)	2 (1–3)	2.13 (1.13)	2 (1–3)	1.81	0.070
Psychological distress before COVID-19	No	1.84 (1.12)	1 (1–2)	1.81 (1.02)	1 (1–2)	0.20	0.845
	Yes	2.63 (1.23)	3 (2–3)	2.64 (1.19)	3 (2–3)	0.32	0.749
International students	No	2.26 (1.24)	2 (1–3)	2.24 (1.18)	2 (1–3)	0.06	0.948
	Yes	2.3 (1.26)	2 (1–3)	2.3 (1.21)	2 (1–3)	0.29	0.771
Total		2.28 (1.24)	2 (1–3)	2.27 (1.19)	2 (1–3)	0.12	0.904

### 3.3. Inter-frequencies analysis of anxiety, stress, and depression levels

For anxiety, stress, and depression levels, it was found that females reported more moderate anxiety (43.3%), whereas males reported increased mild anxiety (66.9%). Students pursuing dentistry reported more anxiety (61.3%) than students pursuing medicine, pharmacy, and physiotherapy. Participants reported normal distress when the course load was 5 to <10 h per week (69.5%). Students experienced moderate anxiety during COVID-19 (53.9%). Students pursuing dentistry reported mild stress (67.7%) compared to other disciplines. Students with a prior mental health issue reported having moderate stress (55.8%) more than students with no history of mental health issues (Table 3).

**Table 3:** Inter-frequencies analysis between initial and current levels of anxiety, stress, and depression using Chi-Square test^*^.

Parameter		Initial frequency *N* (%)				Current frequency *N* (%)			
		Normal/mild	Moderate/more	Chi-square	*p*	Normal/mild	Moderate/more	Chi-square	*p*
Anxiety									
Gender	Female	175 (54.9)	144 (45.1)	2.919	0.088	181 (56.7)	138 (43.3)	4.223	0.04
	Male	90 (63.4)	52 (36.6)			95 (66.9)	47 (33.1)		
Field of study	Medicine	171 (60.9)	110 (39.1)	14.808	0.005	165 (58.7)	116 (41.3)	5.728	0.22
	Dentistry	24 (38.7)	38 (61.3)			45 (72.6)	17 (27.4)		
	Pharmacy	21 (72.4)	8 (27.6)			18 (62.1)	11 (37.9)		
	Physiotherapy	10 (43.5)	13 (56.5)			12 (52.2)	11 (47.8)		
	other	39 (59.1)	27 (40.9)			36 (54.5)	30 (45.5)		
Weekly course load (hrs.)	5 to <10	41 (69.5)	18 (30.5)	7.4	0.06	38 (64.4)	21 (35.6)	2.999	0.392
	10 to <15	68 (52.3)	62 (47.7)			81 (62.3)	49 (37.7)		
	15 to <20	63 (52.1)	58 (47.9)			75 (62)	46 (38)		
	≥20	93 (61.6)	58 (38.4)			82 (54.3)	69 (45.7)		
Year of study (grade)	< 4	188 (58.8)	132 (41.3)	0.686	0.407	186 (58.1)	134 (41.9)	1.326	0.25
	≥ 4	77 (54.6)	64 (45.4)			90 (63.8)	51 (36.2)		
Psychological distress before COVID-19	No	148 (71.5)	59 (28.5)	30.189	<0.001	163 (78.7)	44 (21.3)	55.705	<0.001
	Yes	117 (46.1)	137 (53.9)			113 (44.5)	141 (55.5)		
International students	No	152 (57.4)	113 (42.6)	0.004	0.95	159 (60)	106 (40)	0.004	0.947
	Yes	113 (57.7)	83 (42.3)			117 (59.7)	79 (40.3)		
Stress									
Gender	Female	163 (51.1)	156 (48.9)	2.565	0.109	170 (53.3)	149 (46.7)	2.534	0.111
	Male	84 (59.2)	58 (40.8)			87 (61.3)	55 (38.7)		
Field of study	Medicine	147 (52.3)	134 (47.7)	0.828	0.935	149 (53)	132 (47)	6.988	0.137
	Dentistry	36 (58.1)	26 (41.9)			42 (67.7)	20 (32.3)		
	Pharmacy	15 (51.7)	14 (48.3)			19 (65.5)	10 (34.5)		
	Physiotherapy	13 (56.5)	10 (43.5)			10 (43.5)	13 (56.5)		
	other	36 (54.5)	30 (45.5)			37 (56.1)	29 (43.9)		
Weekly course load (hrs.)	5 to <10	33 (55.9)	26 (44.1)	4.739	0.192	35 (59.3)	24 (40.7)	3.497	0.321
	10 to <15	71 (54.6)	59 (45.4)			80 (61.5)	50 (38.5)		
	15 to <20	55 (45.5)	66 (54.5)			64 (52.9)	57 (47.1)		
	≥20	88 (58.3)	63 (41.7)			78 (51.7)	73 (48.3)		
Year of study (grade)	< 4	173 (54.1)	147 (45.9)	0.098	0.754	170 (53.1)	150 (46.9)	2.919	0.088
	≥ 4	74 (52.5)	67 (47.5)			87 (61.7)	54 (38.3)		
Psychological distress before COVID-19	No	137 (66.2)	70 (33.8)	23.998	<0.001	151 (72.9)	56 (27.1)	45.046	<0.001
	Yes	110 (43.3)	144 (56.7)			106 (41.7)	148 (58.3)		
International students	No	148 (55.8)	117 (44.2)	1.291	0.256	152 (57.4)	113 (42.6)	0.655	0.418
	Yes	99 (50.5)	97 (49.5)			105 (53.6)	91 (46.4)		
Depression									
Gender	Female	186 (58.3)	133 (41.7)	0.357	0.55	189 (59.2)	130 (40.8)	0.197	0.657
	Male	87 (61.3)	55 (38.7)			81 (57)	61 (43)		
Field of study	Medicine	175 (62.3)	106 (37.7)	7.644	0.106	161 (57.3)	120 (42.7)	1.633	0.803
	Dentistry	31 (50)	31 (50)			40 (64.5)	22 (35.5)		
	Pharmacy	21 (72.4)	8 (27.6)			18 (62.1)	11 (37.9)		
	Physiotherapy	11 (47.8)	12 (52.2)			12 (52.2)	11 (47.8)		
	other	35 (53)	31 (47)			39 (59.1)	27 (40.9)		
Weekly course load (hrs.)	5 to <10	32 (54.2)	27 (45.8)	5.924	0.115	36 (61)	23 (39)	4.333	0.228
	10 to <15	75 (57.7)	55 (42.3)			77 (59.2)	53 (40.8)		
	15 to <20	65 (53.7)	56 (46.3)			78 (64.5)	43 (35.5)		
	≥20	101 (66.9)	50 (33.1)			79 (52.3)	72 (47.7)		
Year of study (grade)	< 4	195 (60.9)	125 (39.1)	1.279	0.258	183 (57.2)	137 (42.8)	0.822	0.365
	≥ 4	78 (55.3)	63 (44.7)			87 (61.7)	54 (38.3)		
Psychological distress before COVID-19	No	157 (75.8)	50 (24.2)	43.004	<0.001	157 (75.8)	50 (24.2)	46.215	<0.001
	Yes	116 (45.7)	138 (54.3)			113 (44.5)	141 (55.5)		
International students	No	158 (59.6)	107 (40.4)	0.042	0.838	162 (61.1)	103 (38.9)	1.688	0.194
	Yes	115 (58.7)	81 (41.3)			108 (55.1)	88 (44.9)		

^*^Tests are adjusted for all pairwise comparisons within a row of each innermost subtable using the Bonferroni correction.

### 3.4. Psychological distress predictors

The shift to online education was associated only with the reported stress measurement. More specifically students reported a concern about the ability to build rapport with teachers or professors and this was associated with anxiety and stress. On the other hand, there was a positive correlation between anxiety, stress, and depression and deteriorating sleep quality (Spearman r_anxiety_,=0.325, r_stress_ = 0.410, r_depression_ = 0.440, *p* < 0.001), the feeling of worsening relationships with family and peers (r_anxiety_ = 0.325, r_stress_ = 0.343, r_depression_ = 0.379, p < 0.001), and the sense of loss of efficient time management (r_anxiety_ = 0.321, r_stress_ = 0.345, r_depression_ = 0.410, p < 0.001). Moreover, anxiety, stress, and depression were associated with the need for mental health resources or aid, distress from conversation or implications of COVID-19, the feeling of deteriorating health and tiredness from spending time in front of the screen, the concerns about the current political situation in Eastern Europe, the impact on the future or career, romantic relationships, building sufficient professional competence by remote learning, and lowering of the standard of living, due to the COVID-19 pandemic (Table 4).

**Table 4:** Spearman rank order correlations between psychological distress predictors and anxiety, stress and depression.

Question	Anxiety		Stress		Depression	
	Spearman r	*p*	Spearman r	*p*	Spearman r	*p*
Do you feel the current political situation in Eastern Europe has added on to your stress previously felt by COVID-19?	0.178	<0.001	0.169	<0.001	0.154	0.001
My future/career may be affected due to the implications of COVID-19 pandemic	0.135	0.004	0.149	0.001	0.202	<0.001
Are you concerned about “The new normal,” which is moving towards online education?	0.030	0.527	0.099	0.034	0.070	0.134
I am worried about my family and friends contracting COVID-19	−0.001	0.981	0.003	0.948	−0.018	0.696
I am worried if I can connect with teachers or professors like I used to do before pandemic	0.134	0.004	0.110	0.018	0.080	0.086
I feel mentally drained or exhausted due to conversations or implications of COVID-19	0.222	<0.001	0.239	<0.001	0.274	<0.001
I would like to receive mental health resources or aid to cope up with stress related	0.355	<0.001	0.299	<0.001	0.346	<0.001
In the last month, how often have you felt your health is deteriorating due to the increased time spent at home?	0.246	<0.001	0.293	<0.001	0.378	<0.001
In the last month, how often have you noticed your sleep quality was deteriorated?	0.325	<0.001	0.410	<0.001	0.440	<0.001
In the last month, how often did you do exercise?	0.001	0.987	−0.015	0.740	−0.047	0.316
In the last month, how often have you felt your relationships with family and peers are getting worse?	0.325	<0.001	0.343	<0.001	0.379	<0.001
In the last month, how often have you been worried about your romantic relationships because of the COVID-19 pandemic?	0.168	<0.001	0.236	<0.001	0.235	<0.001
In the last month, how often have you been worried you will not gain sufficient professional competence because of remote learning?	0.172	<0.001	0.198	<0.001	0.235	<0.001
In the last month, how often have you been afraid of lowering your or your family’s standard of living because of the pandemic?	0.191	<0.001	0.162	<0.001	0.232	<0.001
In the last month, how often have you felt you do not manage your time effectively?	0.321	<0.001	0.345	<0.001	0.410	<0.001
In the last month, how often have you felt tired of spending too much time in front of the screen?	0.140	0.003	0.202	<0.001	0.206	<0.001

## 4. Discussion

Due to the transition from online to regular classes and the instability of the political situation in Eastern Europe, anxiety levels have increased significantly compared to the initial levels reported at the beginning of COVID-19. Overall, stress and depression levels remained alarming (mild to moderate), and stress levels were substantially higher in Polish than in international students.

### 4.1. Gender and age

We found that females reported more “moderate/more” anxiety than the male gender (Table 3); however, the comparison of the median was close to being statistically significant (*p* = 0.051). In a systematic review with a sample of 19 studies with a total of 93,569 participants, it was found that females (*p <* 0.05) were highly likely to develop mental distress compared to their male gender counterparts (37, 38).

Regarding age, we did not notice differences between the ages as the students were within the same age range. Sansgiry and Sail reported different results in a cross-sectional study with 1,009 volunteer preclinical medical students (39). Student age was significantly correlated with (*r* = 0.91, *p* < 0.001) anxiety – younger students reported lower test anxiety than older students (39).

### 4.2. Courseload and year of study

Our study noticed that current anxiety and stress levels were significantly improved (decreased) but only in students in the 4th year and higher (Table 2). Fear and anxiety of academic year loss is the main factor for mental distress during COVID-19 (40). Students who reported perceived stress described more academic problems than students who did not report any stress (χ^2^ = 24.162, df = 1, *p* < 0.001) (41). The prevalence of stress was the highest amongst second to third-year medical students (41), as they take their licensing exams and other board exams during this time frame. A correlation analysis by Sansgiry and Sail indicated that students’ perceptions of course load were positively correlated with test anxiety (*r* = 0.24, *p* < 0.001). Students’ time management skills, however, negatively correlated with test anxiety (*r* = −0.20, *p* < 0.001) (39). Factors such as test anxiety levels, student perception of course load, and their ability to manage time are based on their respective academic year (39). However, low levels of stress are essential for better academic performance (42). If the stress is controlled, better achievements can be gained. High levels of stress can disrupt normal functioning. Therefore stress has to be maintained and be in balance. In our research and during the time of the study, students studying 10–20 h per week reported lower levels of anxiety and stress and students studying more than 20 h per week report lower level of depression compared to their initial levels (Table 2).

Concerning the field, students studying dentistry are more likely to experience anxiety (61.3%) and stress (67.7%) compared to students of medicine, pharmacy, and physiotherapy. This might be strongly associated with missing the clinical practice experience since the main clinics for dental practice were closed during the pandemic, which can be a stressor for students fearing a lack of practical knowledge. In contrast, there was no significant difference in the symptoms of depression between different disciplines.

### 4.3. Positive history of psychological distress and COVID-19 implications

In our sample, we noticed that those with a history of psychological distress did not show a significant worsening in their symptoms prepandemic versus at the time of data collection. Only those who did not have such a history experienced a significant decline in anxiety and stress from the initial level during the COVID-19 pandemic (Table 2). They also displayed lower anxiety, stress, and depression (Table 3). To evaluate this point precisely, we need to assess more parameters, such as the usage of psychological consultation, medications, or stimulants by participants with a mental health medical history and the extent of the social support or the volunteer works that all the participants were engaging in.

Concerning distress from conversations or implications of COVID-19, a study of 698 participants in an online survey reported that mediated and interpersonal information exposure had minimal effects on the perceived risk of the COVID-19 pandemic (43). Our results did not agree with the previous research; we found that the conversations on the implications of COVID-19 negatively impacted students’ anxiety, stress, and depression (Table 4). However, the participants in the previous study were the general public, while the participants of our study were medical students who have a medical background and knowledge. The difference in knowledge or direct impact on their practice could cause more concern in our participants.

### 4.4. International students

In our study, 42.5% of the participants were international students. There is no significant difference in the perceived distress between Local and International students. They faced the same distress as the students belonging to Poland. However, Polish students had reported significant improvement in anxiety. Studying at PUMS is chargeable for most international students and the part of non-international students, so financial problems may interfere with students’ future careers. Birmingham et al. reported that 28% of 646 American university students worried about their financial status, and 31.9% of 643 students were concerned about making ends meet in the following months (44). In the qualitative descriptive study (*N* = 11), nursing students also indicated financial problems as a source of strain on their families (45).

### 4.5. Lifestyle

#### 4.5.1. Prolonged stay at home

In a study on the subjective deterioration of health of 1954 participants during the COVID-19 pandemic, researchers reported that the decline of both physical and psychological health was associated with more general anxiety (physical: OR = 1.989, *p* < 0.001; psychological_adjusted_ OR = 1.643, *p* < 0,001) (46). We found similar results and observed a correlation between feelings of deteriorating health and experiencing anxiety, stress, and depression (Table 4). As the previous study also reported, students at PUMS who perceived a higher level of social support were less likely to report a deterioration of physical and psychological health during the COVID-19 pandemic (46). It is essential to provide the appropriate social support programs for this population.

#### 4.5.2. Sleep quality

In a study on the effects of quarantine on sleep quality and psychological distress in 2291 participants, 57.1% reported a deterioration of sleep quality, 32.1% had high anxiety, and 41.8% had high distress linked to COVID-19 (16). In line with these findings, our found a relationship between sleep quality deterioration and anxiety, stress, and depression (Table 4). This confirmed the significance of sound sleep in maintaining good mental health in our population of healthcare students.

#### 4.5.3. Frequency of exercise

The role of exercise in maintaining good mental health during the COVID-19 pandemic was explored by a longitudinal study of 66 college students. It indicated that physical activity directly alleviated general negative emotions (β = −0.12, 95% CI:-0.22, −0.01) as well as depression (*p* = 0.040) (47). Our study, however, did not find any relationship between exercise frequency and psychological distress (Table 4). As the previous study took place at the beginning of the COVID-19 pandemic, our participants could have already adapted to the effect of the pandemic restrictions on the ability to exercise at the ending phase.

#### 4.5.4. Screen time

In a study by Ge et al. (*N* = 1,137), female students who spent 6 or more hours a day in front of a screen experienced higher stress levels than students whose screen time was lower (OR = 1.557, *p* = 0.007) (48). Opdal et al. found that time spent in front of the screen is positively related to mental distress levels in adolescents (*N* = 686, β = 0.038, *p* = 0.008) (49). More than 83% of Indian medical students reported feeling uncomfortable with high levels of screen time during remote learning (50). Our data agree with previous research; spending too much time in front of the screen was positively correlated with students’ anxiety, stress, and depression levels (Table 4). We recommend that students spend as little time in front of the screen outside their classes as possible, especially limiting time spent on social media.

### 4.6. Influence of stationary learning and online learning on psychological distress

In our study, there is no significant association with the concern of moving towards online education for anxiety and depression, but it showed a significant association with the level of stress (*p* < 0.05). In a study involving 804 Polish medical students, 69% of the students preferred the home learning environment, 69% valued unlimited access to resources at home, and 64% highlighted the importance of self-paced learning (51). The responses demonstrated no statistical difference between in-person and remote learning in terms of one’s ability to increase knowledge (*p* = 0.46) (51). However, remote learning was considered less effective than in-person in terms of growing skills (*p* < 0.001), being less interactive in terms of discussion (*p* < 0.001), and providing fewer opportunities to develop social skills (*p* < 0.001) (51). Another cross-sectional study on medical students in Saudi Arabia found that 475 out of 1,288 medical students felt that online tools were insufficient to facilitate remote learning of medical sciences (52). In a study conducted by Khawar et al. in Pakistan (*N* = 2,220), 41% of students experienced severe psychological distress during online learning, and 65% of students experienced dissatisfaction with online learning. Psychological distress predicted dissatisfaction with online classes (Pearson’s r = −0.833). Female students (OR = 1.936, *p* < 0.001) and students older than 30 years (OR = 5.457, *p* < 0.001) experienced dissatisfaction with online courses less often (13). Nishimura et al. presented a study on 473 Japanese medical students, where 29,8% suffered from increased psychological distress because of the transition to remote learning, which they perceived as less effective than stationary education. This subpopulation had larger odds of developing anxiety and depression (OR = 1.97, *p* = 0.009) (53). Almost 71% of American students declared it was harder to focus on their studies during remote learning than during stationary classes (*N* = 645), and 75.2% of them had problems with finding the motivation to study more often (*N* = 644) (44). Students reported a lot of sources of stress during remote learning, e.g., problems with a reliable internet connection, especially during exams, poor contact with colleagues, fear of academic delay, impossibility of learning medical procedures, and inadequate methods used by academic teachers to prevent cheating during online exams (45). Birmingham et al. suggested a transition to stationary learning might cause anxiety in college students (44). Our study was performed when the majority of Polish medical universities had already come back or were coming back to stationery education. Birmingham et al. suggested a transition back to stationary learning might cause anxiety in college students (44).

### 4.7. Impact of war or political instability on students

Anxiety, stress, and depression were significantly associated with students’ political instability in Eastern Europe (*r* = 0.178, *r* = 0.169, and 0.154, respectively; Table 4). A study was conducted on 591 students in the Czech Republic, whereby 63.8% were in medical schools and already following the news about the Russian-Ukrainian war. Thiry-4 % of the study participants expressed anxiety, and 40.7% said they suffered from depression due to European instability (54). Another study was conducted in Italy with over 520 participants to study aggressive reactions associated with the Russian-Ukrainian war and evaluate the effect of emotional cognition, including conflict-related emotions such as anger and shame. The results showed that the aggressive reactions score was significantly correlated with negative emotions like anger and shame (aggressive reactions mean = 2.28, SD = 0.85, anger mean = 3.04, SD = 1.28, and shame mean = 2.33, SD = 1.46) (55). A cross-sectional study conducted on 2,430 Libyan students during a civil war using the General Anxiety Disorder-7 scale showed 64.5% of participants to have varying levels of anxiety (37.5% mild, 16% moderate, and 11% severe) associated with displacement from their homes (*p* < 0.05) (56). Another cross-sectional study of 350 Syrian students used the depression, anxiety, and stress scale and showed a 60.6% prevalence of depression, 35.1% of anxiety, and 52.6% of stress (57). Another study on 1,369 students in war-stricken Syria showed that 53% suffered from post-traumatic stress disorder, and 62% dealt with problematic anger issues (58). Political instability, such as a civil war or genocide, can cause significant distress and have a long-lasting impact on mental health. During a pandemic such as COVID-19, a substantial source of psychological stress, other traumatic events can synergistically lead to a very challenging time for the individual. We must prepare how to respond to and address such intense mental health challenges within the student population across the globe.

### 4.8. Relationship with families, peers, and teachers during studies and distress

We found that worsening relationships with family, friends and romantic partners were correlated with significantly increased anxiety, stress, and depression (Table 4). This shows that good relationships protect students from mental health problems, which can be connected with higher perceived social support, as Z. Li et al. observed in a study involving 224 medical students (11). They showed that social support protected students against psychological distress during the pandemic (β = −0.175, *p* < 0.001) (11). Students with worse relationships with family and friends were at a higher risk of social isolation and mental health problems (59). On the contrary, Chinese students with well-functioning families experienced lower psychological distress during the COVID-19 pandemic (*N* = 1,442, OR = 0.43) (12). In a qualitative descriptive study (*N* = 11), nursing students reported they could spend more time with their families during the pandemic. Still, it also caused increased stress and strain, e.g., taking care of younger siblings or financial problems. Students also felt isolated from their peers and could not share ideas with them. Problems with contact with academic teachers were also reported as a source of stress for nursing students (45).

Moreover, due to the shift to a remote learning environment, it was expected that students would be worried about building rapport with teachers or professors. The study on 224 nursing students about the impact of remote education during COVID-19 reported that 77.6% experienced effectiveness in building rapport with teachers (60). We did not find a similar result; the concern about building rapport with teachers or professors negatively impacted students’ anxiety and stress (Table 4). The previous study on nursing students also reported that the new remote learning system was contemporary (59.3%), had high-quality audio-recording PowerPoint presentations (93.3%) and videos (61.6%), and provided student self-testing (91.1%) (60). The quality of the remote learning system and its contents are other factors that need to be evaluated.

## 5. Strength and limitations

We conducted a cross-sectional study that reached the estimated sample size and showed a significant change in distress levels associated with the COVID-19 pandemic and political instability in Eastern Europe. To our knowledge, it is the first study assessing the psychological distress among healthcare students in Poland after two years of the COVID-19 pandemic after the removal of almost all public health restrictions and during the Ukrainian war. However, we wish to stress some limitations of our study. The measures were made retrospectively, increasing the risk of recall bias. We also used single-item measures of psychological concepts, however, by doing so it was less challenging to collect data from a representative sample. We did not collect data about differential diagnosis such as “long COVID-19” or “post COVID-19.” Also, psychological stress stems from a vast variety of factors, such as problems living away from their hometown, income, cultural competencies, and communication problems, and are not just limited to the ones that our paper states. Using our survey, our paper focused on specific factors to categorize the variables in focus that seemed to be larger components contributing to the psychological stress that the students felt during that specific time. In this type of research, we understand that there is a margin for extraneous variables, but the study focused on specific ones after extensive research on our demographic at that point in time. Moreover, the study is based on self-reporting, therefore the declarations could not be confirmed on the objective ground. Although the questionnaire used in this study demonstrated good reliability and face validity results, it did not fare well in the confirmatory factor analysis suggesting further studies on the same population using validated psychological instruments. Even though our sample size was representative of healthcare students, it was gathered from only one university on the western side of Poland. We may expect different results from universities closer to the Ukrainian border on the eastern side of Poland. The participants were healthcare students, which means there could be a higher awareness of mental illness/distress, so it could be that our respondents have better-adapting skills than other students.

## 6. Conclusion

Our results demonstrated the influence of the Ukrainian war and COVID-19 on anxiety, stress, and depression within a population of healthcare students. Females showed a decrease in reported anxiety levels as time passed, but the levels remained alarming at the time of study. Stress and depression levels remained comparable to their initial levels for both genders (when COVID-19 was first discovered versus post-pandemic). Further research is required to address healthcare students’ coping skills and identify other factors associated with anxiety, stress, and depression, such as socioeconomic status, grades, family size, personal relationships, and cultural background. We recommend that medical universities create, maintain, and improve psychological support programs accessible to all students.

## Data availability statement

The original contributions presented in the study are included in the article/Supplementary material, further inquiries can be directed to the corresponding authors.

## Author contributions

MA and AA: conceptualization, methodology, resources, writing—review and editing, and supervision. MA: validation, formal analysis, and visualization. MA, TH, SN, EM, and PS: investigation and data curation. TH, SN, EM, and PS: writing—original draft preparation. AA: project administration. All authors contributed to the article and approved the submitted version.

## Funding

This work was supported by Poznan University of Medical Sciences for funding the publication cost.

## Conflict of interest

The authors declare that the research was conducted in the absence of any commercial or financial relationships that could be construed as a potential conflict of interest.

## Publisher’s note

All claims expressed in this article are solely those of the authors and do not necessarily represent those of their affiliated organizations, or those of the publisher, the editors and the reviewers. Any product that may be evaluated in this article, or claim that may be made by its manufacturer, is not guaranteed or endorsed by the publisher.
