# Supplementary material for: Psychological distress among healthcare students in Poland from COVID-19 to war on Ukraine: a cross-sectional exploratory study
Source: Front Public Health. 2023 Jun 19;11:1186442. doi: 10.3389/fpubh.2023.1186442 (PMC10315478; doi:10.3389/fpubh.2023.1186442)
Supplement: Supplementary file 2 [file Data_Sheet_2.docx]

| **Demographic data** | |
| --- | --- |
| Age |  |
| Gender |  |
| Field of study |  |
| Year of Study |  |
| What kind of course load have you had since COVID-19 started? | 5 to <10 hours a week  10 to <15 hours a week  15 to <20 hours a week  ≥20 hours a week |
| Are you an international student? | Yes  No |
| Have you struggled with psychological distress prior to the COVID-19 pandemic? | Yes  No |
| **Anxiety, stress and depression scale** | |
| On a scale of 1 to 5, please estimate your anxiety level (1 being Normal and 5 being extremely severe)  Anxiety: If you have an anxiety disorder, you may experience:  Fear, panic or anxiety in situations where most people would not feel anxious or threatened  A constant nagging worry or anxiousness  Sudden panic or anxiety attacks without any clear trigger | Normal  Mild  Moderate  Severe  Extremely severe |
| On a scale of 1 to 5, please estimate your stress level (1 being Normal and 5 being extremely severe)  Stress affects a lot of people and can influence your health. Symptoms include:  Headaches  High blood pressure  Chest pain  Heart palpitations  Skin rashes  Loss of sleep  Stress typically goes away when the stressors disappear | Normal  Mild  Moderate  Severe  Extremely severe |
| On a scale of 1 to 5, please estimate your Depression level (1 being Normal and 5 being extremely severe)  Depression: When you’re depressed, it affects just about everything in your life — how you think, feel, behave and function. You may experience one or more of these symptoms:  Discouragement  Sadness  Hopelessness  Anger  Lack of motivation or interest in life in general  Low energy level  Insomnia  Feeling overwhelmed by daily tasks and personal interactions | Normal  Mild  Moderate  Severe  Extremely severe |
| **Predictors of psychological distress** | |
| Do you feel the current political situation in Eastern Europe has added to your stress previously felt by COVID-19? | Strongly disagree  Disagree  Neutral  Agree  Strongly agree |
| My future/career may be affected due to the implications of the COVID-19 pandemic | Strongly disagree  Disagree  Neutral  Agree  Strongly agree |
| Are you concerned about “The new normal”, which is moving towards online education? | Strongly disagree  Disagree  Neutral  Agree  Strongly agree |
| I am worried about my family and friends contracting COVID-19 | Strongly disagree  Disagree  Neutral  Agree  Strongly agree |
| I am worried if I can connect with teachers or professors like I used to do before the pandemic | Strongly disagree  Disagree  Neutral  Agree  Strongly agree |
| I feel mentally drained or exhausted due to conversations or implications of COVID-19 | Strongly disagree  Disagree  Neutral  Agree  Strongly agree |
| I would like to receive mental health resources or aid to cope with stress related to COVID-19 | Strongly disagree  Disagree  Neutral  Agree  Strongly agree |
| In the last month, how often have you felt your health is deteriorating due to the increased time spent at home? | Never  Rarely  Sometimes  Fairly often  Very often |
| In the last month, how often have you noticed your sleep quality has deteriorated? | Never  Rarely  Sometimes  Fairly often  Very often |
| In the last month, how often did you do exercise? | Never  Rarely  Sometimes  Fairly often  Very often |
| In the last month, how often have you felt your relationships with family and peers are getting worse? | Never  Rarely  Sometimes  Fairly often  Very often |
| In the last month, how often have you been worried about your romantic relationships because of the COVID-19 pandemic? | Never  Rarely  Sometimes  Fairly often  Very often |
| In the last month, how often have you been worried you will not gain sufficient professional competence because of remote learning? | Never  Rarely  Sometimes  Fairly often  Very often |
| In the last month, how often have you been afraid of lowering your or your family's standard of living because of the pandemic? | Never  Rarely  Sometimes  Fairly often  Very often |
| In the last month, how often have you felt you do not manage your time effectively? | Never  Rarely  Sometimes  Fairly often  Very often |
| In the last month, how often have you felt tired of spending too much time in front of the screen? | Never  Rarely  Sometimes  Fairly often  Very often |
