# Supplementary material for: Psychological distress among healthcare students in Poland from COVID-19 to war on Ukraine: a cross-sectional exploratory study
Source: Front Public Health. 2023 Jun 19;11:1186442. doi: 10.3389/fpubh.2023.1186442 (PMC10315478; doi:10.3389/fpubh.2023.1186442)
Supplement: Supplementary file 3 [file Data_Sheet_3.docx]

**“Psychological distress among medical students in Poland; from COVID-19 to War on Ukraine” - a questionnaire validation**

1. **Internal consistency**

|  | Iteam | Source | Mean | SD | alpha |
| --- | --- | --- | --- | --- | --- |
| Q1 | Do you feel the current political situation in Eastern Europe has added on to your stress previously felt by COVID-19? | Pfefferbaum, Betty, Phebe Tucker, Elana Newman, Summer D. Nelson, Pascal Nitiéma, and Rose L. Pfefferbaum. „Terrorism Media Effects in Youth Exposed to Chronic Threat and Conflict in Israel”. Current Psychiatry Reports 21, vol. 4: 28.<https://doi.org/10.1007/s11920-019-1005-4>. | 3.591 | 1.024 | 0.792 |
| Q2 | My future/career may be affected due to the implications of COVID-19 pandemic | Birmingham, Wendy C., Lori L. Wadsworth, Jane H. Lassetter, Tyler C. Graff, Evelyn Lauren, and Man Hung. „COVID-19 Lockdown: Impact on College Students’ Lives”. Journal of American College Health, 1–15.<https://doi.org/10.1080/07448481.2021.1909041>. | 3.563 | 1.045 | 0.791 |
| Q3 | Are you concerned about “The new normal”, which is moving towards online  education? | Wallace, Sharon, Monika S. Schuler, Michelle Kaulback, Karen Hunt, and Manisa Baker. „Nursing Student Experiences of Remote Learning during the COVID‐19 Pandemic”. Nursing Forum 56, vol. 3: 612–18.<https://doi.org/10.1111/nuf.12568>. | 3.424 | 1.034 | 0.796 |
| Q4 | I am worried about my family and friends contracting COVID-19 | Forycka, Joanna, Ewa Pawłowicz-Szlarska, Anna Burczyńska, Natalia Cegielska, Karolina Harendarz, and Michał Nowicki. „Polish Medical Students Facing the Pandemic—Assessment of Resilience, Well-Being and Burnout in the COVID-19 Era”. PLOS ONE 17, vol. 1: | 3.561 | 1.024 | 0.797 |
| Q5 | I am worried if I can connect with teachers or professors like I used to do before pandemic | Wallace, Sharon, Monika S. Schuler, Michelle Kaulback, Karen Hunt, and Manisa Baker. „Nursing Student Experiences of Remote Learning during the COVID‐19 Pandemic”. Nursing Forum 56, vol. 3: 612–18.<https://doi.org/10.1111/nuf.12568>. | 3.217 | 1.063 | 0.787 |
| Q6 | I feel mentally drained or exhausted due to conversations or implications of  COVID-19 | Mheidly, Nour, Mohamad Y. Fares, and Jawad Fares. „Coping With Stress and Burnout Associated With Telecommunication and Online Learning”. Frontiers in Public Health 8: 574969.<https://doi.org/10.3389/fpubh.2020.574969>. | 3.598 | 1.019 | 0.778 |
| Q7 | I would like to receive mental health resources or aid to cope up with stress related to COVID-19 | Forycka, Joanna, Ewa Pawłowicz-Szlarska, Anna Burczyńska, Natalia Cegielska, Karolina Harendarz, and Michał Nowicki. „Polish Medical Students Facing the Pandemic—Assessment of Resilience, Well-Being and Burnout in the COVID-19 Era”. PLOS ONE 17, vol. 1: e0261652.<https://doi.org/10.1371/journal.pone.0261652>. | 3.135 | 1.018 | 0.778 |
| Q8 | In the last month, how often have you felt your health is deteriorating due to the increased time spent at home? | Wang, Peng-Wei, Nai-Ying Ko, Yu-Ping Chang, Chia-Fen Wu, Wei-Hsin Lu, and Cheng-Fang Yen. „Subjective Deterioration of Physical and Psychological Health during the COVID-19 Pandemic in Taiwan: Their Association with the Adoption of Protective Behaviors and Mental Health Problems”. International Journal of Environmental Research and Public Health 17, vol. 18: 6827.<https://doi.org/10.3390/ijerph17186827>. | 3.087 | 1.111 | 0.772 |
| Q9 | In the last month, how often have you noticed your sleep quality was deteriorated? | Gusman, Michaela S, Kevin J Grimm, Adam B Cohen, and Leah D Doane. „Stress and Sleep across the Onset of the Novel Coronavirus Disease 2019 Pandemic: Impact of Distance Learning on US College Students’ Health Trajectories”. Sleep 44, vol. 12: zsab193.<https://doi.org/10.1093/sleep/zsab193>. | 3.133 | 1.232 | 0.777 |
| Q10 | In the last month, how often did you do exercise? | Rivera, Paola Andrea, Bojan Luc Nys, and Fabián Fiestas. „Impact of COVID-19 induced lockdown on physical activity and sedentary behavior among university students: A systematic review”. Medwave 21, vol. 08: e8456–e8456.<https://doi.org/10.5867/medwave.2021.08.8456>. | 2.95 | 1.164 | 0.815 |
| Q11 | In the last month, how often have you felt your relationships with family and peers are getting worse? | Loades, Maria Elizabeth, Eleanor Chatburn, Nina Higson-Sweeney, Shirley Reynolds, Roz Shafran, Amberly Brigden, Catherine Linney, Megan Niamh McManus, Catherine Borwick, and Esther Crawley. „Rapid Systematic Review: The Impact of Social Isolation and Loneliness on the Mental Health of Children and Adolescents in the Context of COVID-19”. Journal of the American Academy of Child & Adolescent Psychiatry 59, vol. 11: 1218-1239.e3.<https://doi.org/10.1016/j.jaac.2020.05.009>. | 2.617 | 1.163 | 0.78 |
| Q12 | In the last month, how often have you been worried about your romantic relationships because of the COVID-19 pandemic? | Yarger, Jennifer, Abigail Gutmann-Gonzalez, Sarah Han, Natasha Borgen, and Martha J. Decker. „Young People’s Romantic Relationships and Sexual Activity before and during the COVID-19 Pandemic”. BMC Public Health 21, vol. 1: 1780.<https://doi.org/10.1186/s12889-021-11818-1>. | 2.439 | 1.389 | 0.782 |
| Q13 | In the last month, how often have you been worried you will not gain sufficient professional competence because of remote learning? | Wallace, Sharon, Monika S. Schuler, Michelle Kaulback, Karen Hunt, and Manisa Baker. „Nursing Student Experiences of Remote Learning during the COVID‐19 Pandemic”. Nursing Forum 56, vol. 3: 612–18.<https://doi.org/10.1111/nuf.12568>. | 3.459 | 1.226 | 0.774 |
| Q14 | In the last month, how often have you been afraid of lowering your or your family's standard of living because of the pandemic? | Lu, Xiaoqian, and Zhibin Lin. „COVID-19, Economic Impact, Mental Health, and Coping Behaviors: A Conceptual Framework and Future Research Directions”. Frontiers in Psychology 12: 759974.<https://doi.org/10.3389/fpsyg.2021.759974>. | 2.413 | 1.162 | 0.778 |
| Q15 | In the last month, how often have you felt you do not manage your time effectively? | Sansgiry, Sujit S., and Kavita Sail. „Effect of Students’ Perceptions of Course Load on Test Anxiety”. American Journal of Pharmaceutical Education 70, vol. 2: 26.<https://doi.org/10.5688/aj700226>. | 3.754 | 1.098 | 0.783 |
| Q16 | In the last month, how often have you felt tired of spending too much time in front of the screen? | Ge, Yinjian, Shimeng Xin, Dechun Luan, Zhili Zou, Xue Bai, Mengting Liu, and Qian Gao. „Independent and Combined Associations between Screen Time and Physical Activity and Perceived Stress among College Students”. Addictive Behaviors 103: 106224.<https://doi.org/10.1016/j.addbeh.2019.106224>. | 3.935 | 1.073 | 0.782 |
|  |  | Cronbach's standardized alpha |  |  | 0.796 |

1. **Face validity**

|  |  | Q1 | Q2 | Q3 | Q4 | Q5 | Q6 | Q7 | Q8 | Q9 | Q10 | Q11 | Q12 | Q13 | Q14 | Q15 | Q16 | total |
| --- | --- | --- | --- | --- | --- | --- | --- | --- | --- | --- | --- | --- | --- | --- | --- | --- | --- | --- |
| Q1 | Pearson Correlation | 1 | .203** | 0.072 | .234** | 0.091 | .229** | .296** | .192** | .159** | 0.016 | .145** | .098* | .144** | .174** | .133** | .149** | .404** |
|  | Sig. (2-tailed) |  | 0.000 | 0.122 | 0.000 | 0.051 | 0.000 | 0.000 | 0.000 | 0.001 | 0.736 | 0.002 | 0.036 | 0.002 | 0.000 | 0.004 | 0.001 | 0.000 |
|  | N | 461 | 460 | 461 | 461 | 461 | 461 | 461 | 461 | 461 | 461 | 461 | 461 | 461 | 461 | 461 | 461 | 461 |
| Q2 | Pearson Correlation | .203** | 1 | .222** | .111* | .198** | .279** | .219** | .181** | .143** | -0.045 | .147** | 0.048 | .237** | .214** | .119* | .153** | .417** |
|  | Sig. (2-tailed) | 0.000 |  | 0.000 | 0.017 | 0.000 | 0.000 | 0.000 | 0.000 | 0.002 | 0.337 | 0.002 | 0.300 | 0.000 | 0.000 | 0.011 | 0.001 | 0.000 |
|  | N | 460 | 460 | 460 | 460 | 460 | 460 | 460 | 460 | 460 | 460 | 460 | 460 | 460 | 460 | 460 | 460 | 460 |
| Q3 | Pearson Correlation | 0.072 | .222** | 1 | 0.013 | .255** | .162** | 0.060 | .161** | .154** | 0.019 | 0.065 | 0.084 | .263** | 0.012 | 0.059 | .155** | .339** |
|  | Sig. (2-tailed) | 0.122 | 0.000 |  | 0.773 | 0.000 | 0.000 | 0.200 | 0.001 | 0.001 | 0.679 | 0.162 | 0.071 | 0.000 | 0.797 | 0.204 | 0.001 | 0.000 |
|  | N | 461 | 460 | 461 | 461 | 461 | 461 | 461 | 461 | 461 | 461 | 461 | 461 | 461 | 461 | 461 | 461 | 461 |
| Q4 | Pearson Correlation | .234** | .111* | 0.013 | 1 | .212** | .149** | .262** | .093* | 0.082 | -0.037 | 0.008 | 0.042 | 0.073 | .273** | 0.066 | .141** | .325** |
|  | Sig. (2-tailed) | 0.000 | 0.017 | 0.773 |  | 0.000 | 0.001 | 0.000 | 0.047 | 0.079 | 0.434 | 0.868 | 0.368 | 0.119 | 0.000 | 0.155 | 0.002 | 0.000 |
|  | N | 461 | 460 | 461 | 461 | 461 | 461 | 461 | 461 | 461 | 461 | 461 | 461 | 461 | 461 | 461 | 461 | 461 |
| Q5 | Pearson Correlation | 0.091 | .198** | .255** | .212** | 1 | .284** | .170** | .285** | .143** | -0.026 | .183** | .184** | .329** | .200** | .114* | .152** | .467** |
|  | Sig. (2-tailed) | 0.051 | 0.000 | 0.000 | 0.000 |  | 0.000 | 0.000 | 0.000 | 0.002 | 0.575 | 0.000 | 0.000 | 0.000 | 0.000 | 0.014 | 0.001 | 0.000 |
|  | N | 461 | 460 | 461 | 461 | 461 | 461 | 461 | 461 | 461 | 461 | 461 | 461 | 461 | 461 | 461 | 461 | 461 |
| Q6 | Pearson Correlation | .229** | .279** | .162** | .149** | .284** | 1 | .440** | .342** | .290** | .124** | .282** | .217** | .268** | .316** | .174** | .254** | .593** |
|  | Sig. (2-tailed) | 0.000 | 0.000 | 0.000 | 0.001 | 0.000 |  | 0.000 | 0.000 | 0.000 | 0.008 | 0.000 | 0.000 | 0.000 | 0.000 | 0.000 | 0.000 | 0.000 |
|  | N | 461 | 460 | 461 | 461 | 461 | 461 | 461 | 461 | 461 | 461 | 461 | 461 | 461 | 461 | 461 | 461 | 461 |
| Q7 | Pearson Correlation | .296** | .219** | 0.060 | .262** | .170** | .440** | 1 | .344** | .288** | 0.048 | .261** | .305** | .236** | .329** | .289** | .217** | .589** |
|  | Sig. (2-tailed) | 0.000 | 0.000 | 0.200 | 0.000 | 0.000 | 0.000 |  | 0.000 | 0.000 | 0.304 | 0.000 | 0.000 | 0.000 | 0.000 | 0.000 | 0.000 | 0.000 |
|  | N | 461 | 460 | 461 | 461 | 461 | 461 | 461 | 461 | 461 | 461 | 461 | 461 | 461 | 461 | 461 | 461 | 461 |
| Q8 | Pearson Correlation | .192** | .181** | .161** | .093* | .285** | .342** | .344** | 1 | .474** | -0.047 | .388** | .303** | .415** | .343** | .362** | .370** | .660** |
|  | Sig. (2-tailed) | 0.000 | 0.000 | 0.001 | 0.047 | 0.000 | 0.000 | 0.000 |  | 0.000 | 0.312 | 0.000 | 0.000 | 0.000 | 0.000 | 0.000 | 0.000 | 0.000 |
|  | N | 461 | 460 | 461 | 461 | 461 | 461 | 461 | 461 | 461 | 461 | 461 | 461 | 461 | 461 | 461 | 461 | 461 |
| Q9 | Pearson Correlation | .159** | .143** | .154** | 0.082 | .143** | .290** | .288** | .474** | 1 | -0.035 | .414** | .266** | .306** | .334** | .354** | .262** | .596** |
|  | Sig. (2-tailed) | 0.001 | 0.002 | 0.001 | 0.079 | 0.002 | 0.000 | 0.000 | 0.000 |  | 0.455 | 0.000 | 0.000 | 0.000 | 0.000 | 0.000 | 0.000 | 0.000 |
|  | N | 461 | 460 | 461 | 461 | 461 | 461 | 461 | 461 | 461 | 461 | 461 | 461 | 461 | 461 | 461 | 461 | 461 |
| Q10 | Pearson Correlation | 0.016 | -0.045 | 0.019 | -0.037 | -0.026 | .124** | 0.048 | -0.047 | -0.035 | 1 | -0.058 | 0.074 | -0.042 | -0.043 | -.137** | -0.006 | .106* |
|  | Sig. (2-tailed) | 0.736 | 0.337 | 0.679 | 0.434 | 0.575 | 0.008 | 0.304 | 0.312 | 0.455 |  | 0.217 | 0.111 | 0.369 | 0.361 | 0.003 | 0.895 | 0.022 |
|  | N | 461 | 460 | 461 | 461 | 461 | 461 | 461 | 461 | 461 | 461 | 461 | 461 | 461 | 461 | 461 | 461 | 461 |
| Q11 | Pearson Correlation | .145** | .147** | 0.065 | 0.008 | .183** | .282** | .261** | .388** | .414** | -0.058 | 1 | .382** | .311** | .324** | .335** | .236** | .570** |
|  | Sig. (2-tailed) | 0.002 | 0.002 | 0.162 | 0.868 | 0.000 | 0.000 | 0.000 | 0.000 | 0.000 | 0.217 |  | 0.000 | 0.000 | 0.000 | 0.000 | 0.000 | 0.000 |
|  | N | 461 | 460 | 461 | 461 | 461 | 461 | 461 | 461 | 461 | 461 | 461 | 461 | 461 | 461 | 461 | 461 | 461 |
| Q12 | Pearson Correlation | .098* | 0.048 | 0.084 | 0.042 | .184** | .217** | .305** | .303** | .266** | 0.074 | .382** | 1 | .362** | .317** | .250** | .300** | .562** |
|  | Sig. (2-tailed) | 0.036 | 0.300 | 0.071 | 0.368 | 0.000 | 0.000 | 0.000 | 0.000 | 0.000 | 0.111 | 0.000 |  | 0.000 | 0.000 | 0.000 | 0.000 | 0.000 |
|  | N | 461 | 460 | 461 | 461 | 461 | 461 | 461 | 461 | 461 | 461 | 461 | 461 | 461 | 461 | 461 | 461 | 461 |
| Q13 | Pearson Correlation | .144** | .237** | .263** | 0.073 | .329** | .268** | .236** | .415** | .306** | -0.042 | .311** | .362** | 1 | .319** | .377** | .305** | .630** |
|  | Sig. (2-tailed) | 0.002 | 0.000 | 0.000 | 0.119 | 0.000 | 0.000 | 0.000 | 0.000 | 0.000 | 0.369 | 0.000 | 0.000 |  | 0.000 | 0.000 | 0.000 | 0.000 |
|  | N | 461 | 460 | 461 | 461 | 461 | 461 | 461 | 461 | 461 | 461 | 461 | 461 | 461 | 461 | 461 | 461 | 461 |
| Q14 | Pearson Correlation | .174** | .214** | 0.012 | .273** | .200** | .316** | .329** | .343** | .334** | -0.043 | .324** | .317** | .319** | 1 | .289** | .211** | .587** |
|  | Sig. (2-tailed) | 0.000 | 0.000 | 0.797 | 0.000 | 0.000 | 0.000 | 0.000 | 0.000 | 0.000 | 0.361 | 0.000 | 0.000 | 0.000 |  | 0.000 | 0.000 | 0.000 |
|  | N | 461 | 460 | 461 | 461 | 461 | 461 | 461 | 461 | 461 | 461 | 461 | 461 | 461 | 461 | 461 | 461 | 461 |
| Q15 | Pearson Correlation | .133** | .119* | 0.059 | 0.066 | .114* | .174** | .289** | .362** | .354** | -.137** | .335** | .250** | .377** | .289** | 1 | .405** | .532** |
|  | Sig. (2-tailed) | 0.004 | 0.011 | 0.204 | 0.155 | 0.014 | 0.000 | 0.000 | 0.000 | 0.000 | 0.003 | 0.000 | 0.000 | 0.000 | 0.000 |  | 0.000 | 0.000 |
|  | N | 461 | 460 | 461 | 461 | 461 | 461 | 461 | 461 | 461 | 461 | 461 | 461 | 461 | 461 | 461 | 461 | 461 |
| Q16 | Pearson Correlation | .149** | .153** | .155** | .141** | .152** | .254** | .217** | .370** | .262** | -0.006 | .236** | .300** | .305** | .211** | .405** | 1 | .542** |
|  | Sig. (2-tailed) | 0.001 | 0.001 | 0.001 | 0.002 | 0.001 | 0.000 | 0.000 | 0.000 | 0.000 | 0.895 | 0.000 | 0.000 | 0.000 | 0.000 | 0.000 |  | 0.000 |
|  | N | 461 | 460 | 461 | 461 | 461 | 461 | 461 | 461 | 461 | 461 | 461 | 461 | 461 | 461 | 461 | 461 | 461 |
| total | Pearson Correlation | .404** | .417** | .339** | .325** | .467** | .593** | .589** | .660** | .596** | .106* | .570** | .562** | .630** | .587** | .532** | .542** | 1 |
|  | Sig. (2-tailed) | 0.000 | 0.000 | 0.000 | 0.000 | 0.000 | 0.000 | 0.000 | 0.000 | 0.000 | 0.022 | 0.000 | 0.000 | 0.000 | 0.000 | 0.000 | 0.000 |  |
|  | N | 461 | 460 | 461 | 461 | 461 | 461 | 461 | 461 | 461 | 461 | 461 | 461 | 461 | 461 | 461 | 461 | 461 |

**. Correlation is significant at the 0.01 level (2-tailed)

*. Correlation is significant at the 0.05 level (2-tailed)
